# Causal influences of neuropsychiatric disorders on Alzheimer’s disease

**DOI:** 10.1038/s41398-024-02822-1

**Published:** 2024-02-23

**Authors:** Ancha Baranova, Qian Zhao, Hongbao Cao, Vikas Chandhoke, Fuquan Zhang

**Affiliations:** 1 School of Systems Biology, George Mason University, Manassas, USA; 2 Research Centre for Medical Genetics, Moscow, Russia; 3 Department of Psychiatry, The Affiliated Brain Hospital of Nanjing Medical University, Nanjing, China; 4 Institute of Neuropsychiatry, The Affiliated Brain Hospital of Nanjing Medical University, Nanjing, China

**Keywords:** Comparative genomics, Psychiatric disorders

## Abstract

Previous studies have observed a significant comorbidity between Alzheimer’s disease (AD) and some other neuropsychiatric disorders. However, the mechanistic connections between neuropsychiatric disorders and AD are not well understood. We conducted a Mendelian randomization analysis to appraise the potential influences of 18 neurodegenerative and neuropsychiatric disorders on AD. We found that four disorders are causally associated with increased risk for AD, including bipolar disorder (BD) (OR: 1.09), migraine (OR: 1.09), schizophrenia (OR: 1.05), and Parkinson’s disease (PD) (OR: 1.07), while attention-deficit/hyperactivity disorder (ADHD) was associated with a decreased risk for AD (OR: 0.80). In case of amyotrophic lateral sclerosis (OR: 1.04) and Tourette’s syndrome (OR: 1.05), there was suggestive evidence of their causal effects of on AD. Our study shows that genetic components predisposing to BD, migraine, schizophrenia, and PD may promote the development of AD, while ADHD may be associated with a reduced risk of AD. The treatments aimed at alleviating neuropsychiatric diseases with earlier onset may also influence the risk of AD-related cognitive decline, which is typically observed later in life.

## Introduction

Neurodegenerative and neuropsychiatric diseases represent differing parts of the spectrum of brain disorders. Typically, neurodegenerative disorders are late-onset and have a progressive clinical course, with clear structural marks of the pathophysiological process developing gradually. Alzheimer’s disease (AD) is a “classical” example of a neurodegenerative disorder. So-called senile plaques and neurofibrillary tangles are regarded as the pathological ‘hallmarks’ of AD, and the aggregation of α-synuclein in Lewy bodies is commonly discussed as the culprit of Parkinson’s disease (PD) [1]. Genome-wide association studies (GWAS) have identified shared risk loci across various pairs of neurodegenerative diseases, such as *APOE* in AD and Lewy body dementia (LBD), or *GBA* and *SNCA* in PD and LBD [2]. Moreover, it has been shown that polygenic risk scores (PRS) for one neurodegenerative disease may predict the risk of another disease. For example, the PRS for PD also predicts the risk for LBD [2].

On the other hand, the nature of neuropsychiatric conditions is more “soft”; these diseases are described as “functional” disorders with an onset in early or middle adulthood and a remitting course, with little or no structurally distinct biomarkers and a possibility of being pharmacologically reversed. These diseases correlate with each other genetically, forming a hierarchical classificatory system [3–5]. While some studies of the genetic relations were performed for neuropsychiatric conditions as a group, a majority of genome-wide investigations of neuropsychiatric and neurodegenerative conditions were either evaluated causality in one particular neurodevelopmental condition paired with a neuropsychological one, i.e., AD and bipolar disorder (BD) [6] or the relationships were explored within only one commonly comorbid nosological group [7, 8].

Some recent studies, however, suggest that genetic relationships among neurodegenerative and neuropsychiatric diseases may form a complex, entangled pattern, with possible involvement of pleiotropic genes and multiple co-regulated or cross-talking pathways [9]. These findings are exemplified by common observations that neurodegenerative diseases may present with comorbid neuropsychiatric symptoms. In recent years, Mendelian randomization (MR) analysis has been frequently used for exploring causal relationships between various diseases at the genetic level [10–13]. In MR, the diseases are represented by genetic variants contributing to particular phenotypes. Therefore, MR results may provide novel clues to disease pathogenesis or plausible explanations for the results of observational studies by evaluating the causality and mutuality of the relationships within a pair of traits. To provide new insights into the shared genetics of neurodegenerative AD and neuropsychiatric conditions, we performed an MR study of the genetic components of common conditions representing both ends of the brain disease spectrum, neuropsychiatric and neurophysiological ones, and AD.

## Methods

### GWAS summary datasets

A total of 19 GWAS summary datasets for the 19 neuropsychiatric disorders were utilized in this study, including AD [14], attention-deficit/hyperactivity disorder (ADHD) [15], alcohol dependence [16], amyotrophic lateral sclerosis (ALS) [17], anorexia nervosa [18], anxiety disorder [19], autism spectrum disorder [20], BD [21], epilepsy [22], insomnia [23], major depressive disorder (MDD) [24], migraine [25], multiple sclerosis (MS) [26], obsessive-compulsive disorder [27], PD [28], posttraumatic stress disorder [29], schizophrenia [30], stroke [31], and Tourette’s syndrome [32] (Table 1). The datasets were obtained from the Psychiatric Genomics Consortium (PGC), GWAS Catalog, and other consortia. Sample sizes ranged from 10,640 to 873,341 for the datasets, with all the participants being of European origin.

**Table 1:** Summary information of the datasets.

Trait	Full name	Authors	Year	PMID	Ncase	Ncontrol	*N*
AD	Alzheimer’s disease	Bellenguez, et al.	2022	35379992	86531	676386	762917
ADHD	Attention deficit/hyperactivity disorder	Demontis, et al.	2023	36702997	38691	275986	292548
Alcohol dependence	Alcohol dependence	Walters, et al.	2018	30482948	11569	34999	46568
ALS	Amyotrophic lateral sclerosis	Nicolas, et al.	2018	29566793	20806	59804	80610
Anorexia nervosa	Anorexia nervosa	Watson, et al.	2019	31308545	16992	55525	72517
Anxiety disorder	Anxiety disorder	Otowa, et al.	2016	26857599	7016	14745	17310
ASD	Autism spectrum disorder	Grove, et al.	2019	30804558	18381	27969	46350
BD	Bipolar disorder	Mullins, et al.	2021	34002096	41917	371549	413466
Epilepsy	Epilepsy	Abou-Khalil, et al.	2018	30531953	15212	29677	44889
Insomnia	Insomnia	Jansen, et al.	2019	30804565	109402	277131	386533
MDD	Major depressive disorder	Howard, et al.	2019	30718901	246363	561190	807553
Migraine	Migraine	Hautakangas, et al.	2022	35115687	102084	771257	873341
MS	Multiple sclerosis	Patsopoulos, et al.	2019	31604244	47429	68374	115803
OCD	Obsessive-compulsive disorder	Arnold, et al.	2017	28761083	2688	7952	10640
PD	Parkinson’s disease	Nalls, et al.	2019	31701892	33674	449056	482730
PTSD	Posttraumatic stress disorder	Nievergelt, et al.	2019	31594949	23212	151447	174659
Schizophrenia	Schizophrenia	Trubetskoy, et al.	2022	35396580	53386	77258	130644
Stroke	Stroke	Malik, et al.	2018	29531354	40585	406111	446696
Tourette’s syndrome	Tourette syndrome	Yu, et al.	2019	30818990	4819	9488	14307

### MR analysis

The MR analyses were accomplished using three methods implemented in the R package TwoSampleMR (version 0.5.6) [33]. MR analysis requires three main assumptions about the instrumental variable (IV): (1) It is closely related to exposure; (2) It is not related to any confounding factors that affected the exposure-outcome association; (3) It does not affect outcomes (except by association with exposure) [34]. The inverse-variance weighted (IVW) model was applied as the main method, while the other two models, weighted median and MR-Egger were utilized as complementary methods for assessment of sensitivity. The intercept of the MR-Egger regression was employed to assess directional pleiotropy [35]. The heterogeneity of the MR associations was gauged by both I^2^ statistics and Cochran’s Q test (both *I*^2^ > 0.25 and *P* < 0.05) [36]. Significant associations were determined by IVW-based *P* values < 0.05. For each MR analysis, genome-wide significant single-nucleotide polymorphisms (*P* < 5 × 10^-8^) in the exposure dataset were selected to derive IVs (r^2^ < 0.01 within a 10 Mb window).

## Results

### MR analysis

**Fig. 1:**
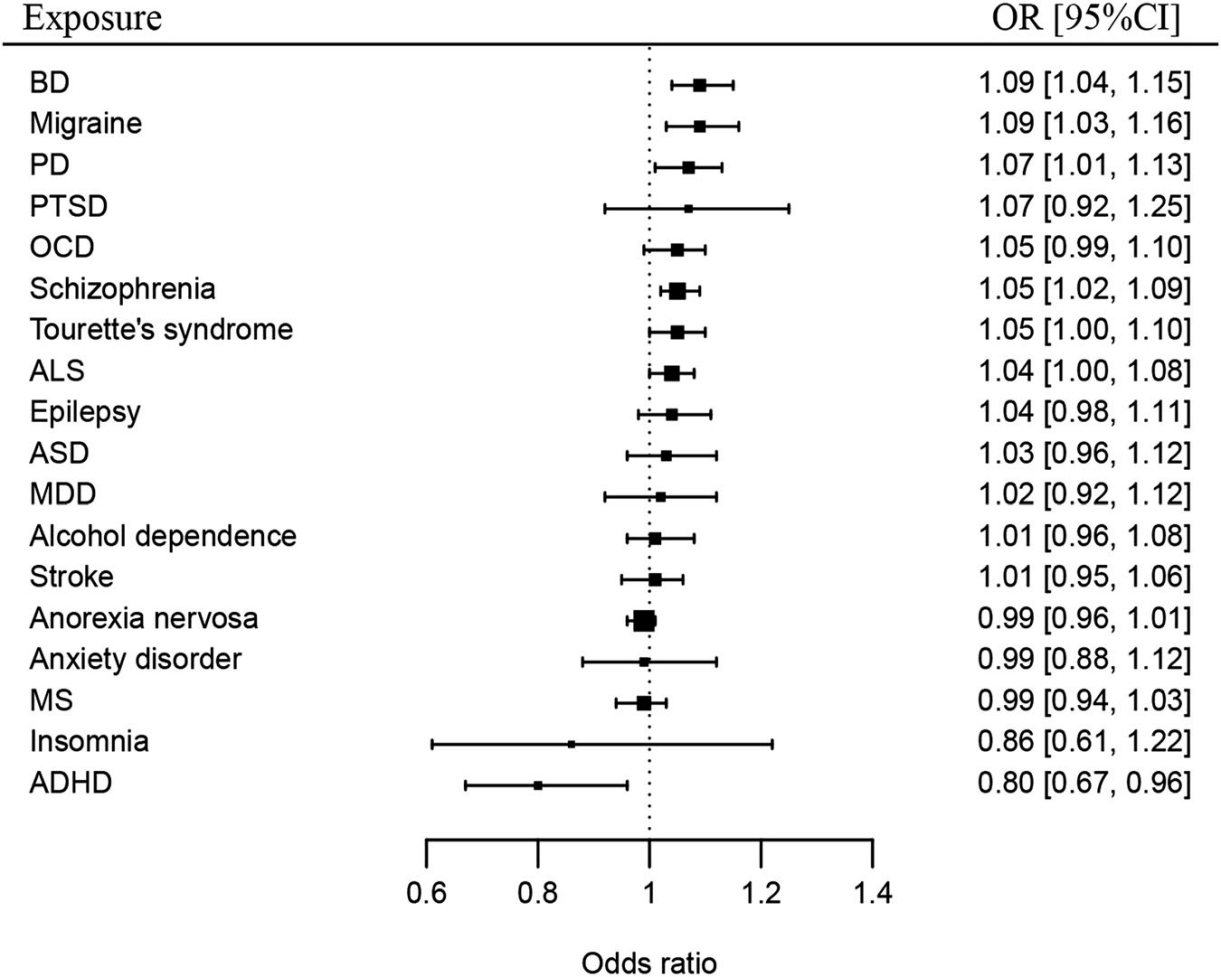
Causal effects of neuropsychiatric disorders on AD. AD Alzheimer’s disease, ADHD attention-deficit/hyperactivity disorder, ALS amyotrophic lateral sclerosis, ASD autism spectrum disorder, BD bipolar disorder, MDD major depressive disorder, MS multiple sclerosis, OCD obsessive-compulsive disorder, PD Parkinson’s disease, PTSD posttraumatic stress disorder.

For each of the neuropsychiatric disorders, their causal effects on AD were evaluated in MR analyses and summarized in Table 2 and Fig. 1. Four disorders were causally associated with an increased risk for AD, including BD (OR: 1.09, CI: 1.04–1.15, *P* = 5.60E-04), migraine (OR: 1.09, CI: 1.03–1.16, *P* = 0.002), schizophrenia (OR: 1.05, CI: 1.02–1.09, *P* = 6.84E-04), and PD (OR: 1.07, CI: 1.01–1.13, *P* = 0.022); while ADHD was associated with a decreased risk for AD (OR: 0.80, CI: 0.67–0.96, *P* = 0.014). Evidence that ALS (OR: 1.04, CI: 1.00–1.08, *P* = 0.053) and Tourette’s syndrome (OR: 1.05, CI: 1.00–1.10, *P* = 0.067) have a causal effect on AD was suggestive. In addition, our results do not support the causal effects of epilepsy (OR: 1.04, CI: 0.98–1.11, *P* = 0.216), MDD (OR: 1.02, CI: 0.92–1.12, *P* = 0.734), MS (OR: 0.99, CI: 0.94–1.03, *P* = 0.611), or stroke (OR: 1.01, CI: 0.95–1.06, *P* = 0.817) on AD.

**Table 2:** Causal effects of the neuropsychiatric disorders on AD.

Exposure	Outcome	b (se)	OR [95% CI]	N_IV	P
ADHD	AD	-0.222 (0.090)	0.80 [0.67-0.96]	26	0.014
Alcohol dependence	AD	0.015 (0.031)	1.01 [0.96-1.08]	25	0.638
ALS	AD	0.039 (0.020)	1.04 [1.00-1.08]	47	0.053
Anorexia nervosa	AD	-0.013 (0.013)	0.99 [0.96-1.01]	92	0.313
Anxiety disorder	AD	-0.010 (0.062)	0.99 [0.88-1.12]	18	0.873
ASD	AD	0.033 (0.040)	1.03 [0.96-1.12]	54	0.412
BD	AD	0.087 (0.025)	1.09 [1.04-1.15]	58	5.60E-04
Epilepsy	AD	0.042 (0.034)	1.04 [0.98-1.11]	78	0.216
Insomnia	AD	-0.149 (0.178)	0.86 [0.61-1.22]	14	0.402
MDD	AD	0.017 (0.049)	1.02 [0.92-1.12]	96	0.734
Migraine	AD	0.089 (0.029)	1.09 [1.03-1.16]	37	2.02E-03
MS	AD	-0.012 (0.023)	0.99 [0.94-1.03]	95	0.611
OCD	AD	0.045 (0.028)	1.05 [0.99-1.10]	22	0.103
PD	AD	0.067 (0.029)	1.07 [1.01-1.13]	25	0.022
PTSD	AD	0.069 (0.080)	1.07 [0.92-1.25]	38	0.387
Schizophrenia	AD	0.053 (0.016)	1.05 [1.02-1.09]	178	6.84E-04
Stroke	AD	0.006 (0.027)	1.01 [0.95-1.06]	78	0.817
Tourette’s syndrome	AD	0.046 (0.025)	1.05 [1.00-1.10]	36	0.067

*AD* Alzheimer’s disease, *ADHD* attention-deficit/hyperactivity disorder, *ALS* amyotrophic lateral sclerosis, *ASD* autism spectrum disorder, *BD* bipolar disorder, *b* MR estimate, *CI* confidence interval, *MDD* major depressive disorder, *MS* multiple sclerosis, *N_IV* number of instrumental variables, *OCD* obsessive-compulsive disorder, *OR* odds ratio, *PD* Parkinson’s disease, *P* *P* value, *PTSD* posttraumatic stress disorder, *se* standard error.

The directions of causal effect estimates revealed in heterogeneity analysis across the set of applied techniques were largely the same (Supplementary Table 1). No directional pleiotropy was detected in the result of the MR-Egger model (*P* > 0.05 and MR-Egger intercept < 0.01). On the other hand, Cochran’s Q test and the I^2^ statistics suggested the heterogeneity for some effect estimates.

## Discussion

This study revealed the causal effects of several neuropsychiatric disorders on AD and suggested the possibility of intersecting pathways spanning the spectrum of neurodegenerative and neuropsychiatric diseases.

Our results suggest that the genetic components of BD, schizophrenia, migraine, and PD may causally contribute to the risk for the development of AD later in life. Two of these conditions, migraine, and PD, belong to the neurodegenerative part of the spectrum, while schizophrenia and BD are typically classified as neuropsychiatric diseases, with a certain degree of intertwining. In a recent PRS-based MR study, schizophrenia was causally linked with higher odds of BD (OR: 1.52, CI: 1.36–1.70) [37], while in another MR study of blood metabolite levels, the odds for either BD (OR: 0.72) or schizophrenia (OR: 0.74) were causally lower when the levels of the amino acid derivative N-acetylornithine were higher [38]. Nondirectional polygenic overlap between AD and BD has been reported before, with the shared loci implicating the *MARK2* and *VAC14* genes as possible culprits [6]. In addition, significant local genetic correlations were detected between schizophrenia and AD as well as PD [9]. Notably, all the observations support recently proposed metabolome and transcriptome-driven models of shared underlining molecular pathobiology of brain illnesses, where the disturbance of the tissue-wide molecular networks promotes aging in general, and AD in particular.

Notably, schizophrenia shares many clinical and pathophysiological features with AD [39]. In a recent meta-analysis, both schizophrenia and AD were associated with accelerated aging of brain tissues, possibly through the common feature of enhanced neuroinflammation [40]. Comparative studies examining mechanisms contributing to both AD and schizophrenia highlight synaptic destruction, as well as the shortening of the telomere length [41–43]. In observational studies, patients with schizophrenia have a significantly higher risk of developing AD when compared with the general population [44]. Studies have demonstrated a clear overlap in white matter defect patterns between schizophrenia and AD, with striking similarities that are both replicable and related to the core cognitive deficits of the respective disorders [45]. It was suggested that psychosis, and especially delusion, which are commonly detected in AD patients, share some of their genetic components with schizophrenia [46]. These findings should be utilized as starting points for assessing mechanistic pathways jointly contributing to AD and schizophrenia as our study implies.

Many studies performed in small, community-based settings have indicated positive effects of migraine history on either all-cause dementia or Alzheimer’s dementia [47]. Notably, migraine is known for its genetic clustering with cardiovascular conditions of the brain, which often complicates diagnostics of dementia in the elderly. Moreover, the causal influence of migraine on different types of brain-damaging cardiovascular conditions may be exerted with opposing signs, thus, complicating the picture by making it dependent on frequencies of cardiovascular events of particular types and in particular populations. For example, in one recent MR study, concordant risks of the migraine and the dissection of the cervical artery were counterbalanced by opposite risk patterns between migraine and large artery stroke [48].

An intersection of AD and PD is often described in the context of LBD, where LBD, not evaluated in the current study, presents as a bridging entity. Notably, previous studies have shown that genome-wide genetic risk scores of AD and PD do not interact in LBD prediction. Therefore, our findings of causal effects exerted on AD by PD are novel. Interestingly, local pleiotropy influencing both of these diseases was found in the *HLA* and *MAPT* loci, as well as within *SBCA* and *CLU*-containing stretches of DNA [9].

In addition, we found that genetic predisposition to ADHD reduces the risk of AD, and the effect was relatively strong (OR: 0.80). The most plausible explanation for the anti-AD effect of the genetic component of ADHD is the overall ADHD-associated increase in propensity to exercise and physical activity. In a recent “meta-umbrella” systematic synthesis of umbrella reviews, robust protective effects against AD were detected for high physical activity, with a hazard ratio of 0.62 [49]. Notably, other studies found that ADHD-predicting PRS (i.e., a genetic component of ADHD phenotype) is associated with increased overall activity, with genetic correlation analysis corroborating the PRS findings [2]. On the other hand, some studies have reported limited evidence for a causal effect of genetic liability to ADHD on AD or an association of genetic liability for ADHD, as measured by ADHD-PRS, with either cognitive decline or the development of AD pathophysiology in elderly individuals, and even with increased cerebrospinal fluid p-tau181 levels, when affected individuals were Aβ-positive [50]. It seems that future studies aimed at the dissection of the ADHD-AD conundrum have to take into account the measurements of overall physical activity and voluntary exercise.

The presented study is not free of limitations. In particular, for each neuropsychiatric or neurodegenerative disease, we have measured only genetic liability, while the effects of the environmental factors were not considered, thus, limiting our conclusion in their scope. Some environmental factors, including the ability to maintain physical activity, are known to influence both neuropsychiatric conditions and AD, and may mediate the associations between selected pairs of conditions. Considering that some datasets contained participants from the UK Biobank, the AD dataset may have some overlapping samples with some of the exposure datasets, including MDD, PD, BD, and migraine. Therefore, the MR estimates observed in this study should be interpreted with caution.

### Supplementary information

Supplementary Table 1. Causal effects of the neuropsychiatric disorders on AD.

## Data Availability

All de-identified data are publicly available.
